# Tuberculosis treatment outcomes of six and eight month treatment regimens in districts of Southwestern Ethiopia: a comparative cross-sectional study

**DOI:** 10.1186/s12879-016-1917-0

**Published:** 2016-11-08

**Authors:** Abyot Asres, Degu Jerene, Wakgari Deressa

**Affiliations:** 1 Department of Public Health, College of Health Sciences, MizanTepi University, Mizan Aman, Ethiopia; 2 Management Science for Health, HEAL TB project, Addis Ababa, Ethiopia; 3 Department of Preventive Medicine, School of Public Health, College of Health Sciences, Addis Ababa University, Addis Ababa, Ethiopia

**Keywords:** Tuberculosis, Continuation phase, treatment regimen, Treatment outcome, Ethiopia

## Abstract

**Background:**

A switch of continuation phase tuberculosis (TB) treatment regimen from Ethambutol (E) and Isoniazid (H) combination for 6 months (6EH) to Rifampicin (R) and Isoniazid (H) combination for 4 months (4RH) was recommended. However, the effect of the regimen switch in Ethiopian setting is not known.

**Methods:**

A comparative cross-sectional study among 790 randomly selected new cases of TB (395 each treated with 4RH and 6EH during the continuation phase) was conducted in nine health centers and one hospital in three zones in southwestern Ethiopia. Data were abstracted from the standard unit TB register composed of standard case and treatment outcome definitions. Data were analyzed using STATA version 13 where binary logistic regression was fitted to identify independent predictors of unsuccessful treatment outcomes at 5 % significance level.

**Results:**

Over all, 695 (88 %) of the patients had a successful treatment outcome with statistically significant difference (85.3 % vs 90.6 %, *p* = 0.02) among the 6HE and 4RH regimens, respectively. After adjusting for confounders, 4RH continuation phase treatment regimen adjusted odds ratio (AOR) [(95 % confidence interval (CI)) 0.55 (0.34,0.89)], age [AOR (95 % CI 1.02 (1.001,1.022)], rural residence [AOR (95 % CI) 2.1 (1.18,3.75)] Human Immunodeficiency virus (HIV) positives [AOR (95 % CI) 2.39 (1.12,5.07)] and increased weight at the end of the second month [AOR (95 % CI 0.28 (0.11,0.72)] independently predicted treatment outcome.

**Conclusion:**

The switch of continuation phase TB treatment regimen from 6EH to 4RH has brought better treatment outcomes which imply applicability of the recommendation in high prevalent and resource constrained settings. Therefore, it should be maintained and augmented through further studies on its impact among the older, rural residents and HIV positives.

**Electronic supplementary material:**

The online version of this article (doi:10.1186/s12879-016-1917-0) contains supplementary material, which is available to authorized users.

## Background

In the history of tuberculosis (TB) control, discovery of chemotherapy [1] brought about dramatic changes in patient survival. Before the advent of chemotherapy, 30–40 % of TB cases used to die within a year and 50–70 % within 5–7 years after the onset of TB illness [2]. Introduction of chemotherapy resulted in cure and reduction of mortality for majority of TB cases [1, 3]. However, shortly after the therapy, resistance to drug and poor adherence by patients were reported [4]. Consequently, the first standard combination therapy for 12 months comprised of Thiacetazone (T), Isoniazid(H) and streptomycin(S) for the first 2 months (2STH) followed by T and H for 10 months(10TH) was issued [2]. Subsequent to the introduction of rifampicin (R), effective short-course chemotherapy regimens for less than 12 months became standard therapy [2, 5–7]. The short-course regimens comprised of an initial, or bactericidal, phase called intensive that aimed to kill bacilli and make patients non infectious and a continuation or sterilizing phase which eliminates persisting mycobacteria to prevent relapse [1, 8]. Thus evidence based combinations of drugs for different categories of cases have been recommended for the two phases across the different regimens [5–7].

Introduction of Rifampicin has shortened TB treatment duration [1, 9]. In 1991, an eight months treatment regimen composed of 2 months intensive with Isoniazid(H), Rifampicin (R), Pyrazinamide(Z) and Ethambutol(E) (2RHZE/S) and 6 months continuation (6HE) phases were recommended for all new cases of TB across the world [6]. To avoid resistance to the most potent drugs, isoniazid and rifampcin and ensure patient adherence, directly observed treatment short course (DOTS) strategy was launched in 1994 [10]. Later in 2003, a directly observed intensive phase treatment followed by two continuation phase regimens, 6 months of isoniazid plus ethambutol (6HE) or 4 months of isoniazid plus rifampicin (4HR) were recommended. The 4HR continuation phase treatment regimen needed to be observed throughout the treatment period whereas the 6EH regimen relied on self administered treatment [5]. As a result, regimens without rifampicin had been considered safer in developing countries owing to irregular treatments and high absentee rates [1, 5]. However, the latest World Health Organization (WHO) guideline recommends 2-month initial phase of (2RHZE) and a 4-month continuation phase (4RH) for the treatment of virtually all forms of new TB cases across the globe [7].

The government of Ethiopia has adopted the switch of 4HR continuation phase TB treatment for all new cases and accommodated in the latest TB treatment guideline [11]. Though global strategies are relevant, investigation of the applicability to the local settings is highly required. A continuation phase treatment with 4HR regimen elsewhere has demonstrated lower unsuccessful treatment outcomes and costs as compared to 6EH continuation phase [12–15]. But well designed studies evaluating effects of the introduction of 4RH for the continuation phase TB treatment in high TB burden and resource limited settings like Ethiopia are limited. Thus, we compared treatment outcomes of TB cases who received 4RH and 6EH continuation phase regimens under routine program condition in high burden and resource limited setting. Our objective was to compare baseline patients’ bacteriologic, socio-demographics, clinical characteristics and treatment outcomes among those TB patients treated with the 4RH and 6EH continuation phase treatment regimens.

## Methods

### Study setting

We conducted this study in ten health facilities (one hospital and nine health centres) in three remote *zones* of Southern Nation Nationalities and Peoples Region (SNNPR), one of the nine regions in Ethiopia with about 18million population [16]. Ethiopia is among the 22 TB High Burden Countries (HBC) where 230,000 incident cases of which 147,592 (64 %) were notified. In the same year, 16100 deaths and 90 % treatment success among the smear positives cases registered in 2011 were reported [17, 18]. The country has adopted and implemented the DOTS strategy for the treatment of all forms of TB. Accordingly new cases of TB had been treated with directly observed RHZE combinations for the first 2 months (2RHZE) followed by self administered EH combinations for six months (6EH) [19]. As of the end of 2011, the continuation phase treatment was switched from 6EH to 4RH. Thus the regimen became directly observed 2RHZE/4RH combinations for all forms of new TB cases throughout the 6 months treatment period [11].

The three study *Zones*, Bench Maji, Kaffa and Sheka are located at the southwestern border of the SNNPR where about 2,064,102 peoples reside [16]. The *zones* (an administrative unit that liaison *weredas* with the region) are organized in to four town administrations and 26 *weredas* (lowest administrative unit closer to the community) those have three hospitals and 65 health centers those provide TB DOTS services for free. However, the three hospitals and only 27 health centers were providing TB/Human Immunodeficeincy Virus (HIV) collaborative activities [20].

### Study design and sampling

A comparative cross-sectional study among TB cases treated with 2RHZE/6EH and 2RHZE/4RH regimens was carried out. New cases registered between 2008 and 2014 were eligible of which those aged above 15 years were studied. Sample size was estimated using the Stat Calc program of Epi Info version 7 [21] with 95 % confidence level, 80 % power and ratio of 6EH to 4RH (*r* = 1). Accordingly, 512 cases (256 from each group) was required to detect 7 % difference [12] in the proportion of unsuccessful outcome among the 6EH and 4RH groups. Finally, considering the design effect of 1.5 and 10 % missing records, a total of 846 cases were required. The samples were selected through proportional allocation to the three zones followed by selection of *weredas* and health facilities from the zone using probability proportional to size. The allocation and selection was made based on total number of cases reported from the *weredas* and health facilities during 2008 through 2014. Lastly, cases from the selected health facilities were selected randomly using SPSS statistical software using TB unit number of the cases.

Data were extracted from a standard unit TB register recommended by the WHO [11, 19, 22] using extraction format prepared for the study. Thus patients’ baseline attributes (age, gender, residence, sputum smear, type of TB, HIV status) and follow-up measures (sputum smear, weight, drug regimen and treatment outcomes) were extracted. The following standard case and outcome definitions were adopted and used for the study [11, 19].

- **New case** of TB a patient who never had treatment for TB, or had been on anti-TB treatment for less than four weeks in the past
- **Other cases** are those patients who do not fulfill the criteria for new, relapse, and return after default or treatment after failure.
- **Cured:** a patient whose sputum smear or culture was positive at the beginning of the treatment but who was smear or culture-negative in the last month of treatment and on at least one previous occasion.
- **Treatment completed:** completed treatment but does not have a negative sputum smear or culture result in the last month of treatment and on at least one previous occasion.
- **Treatment failure:** a patient whose sputum smear or culture is positive at 5 months or later during treatment or patients found to harbor Multidrug Resistant (MDR) TB strain at any point of time during the treatment, whether they are smear-negative or -positive.
- **Died:** a patient who dies for any reason during the course of TB treatment.
- **Defaulter/loss to follow-up:** a patient who has been on treatment for at least 4 weeks and interrupted treatment for eight or more consecutive weeks.
- **Successful treatment:** a treatment that ends up in cure or treatment completion
- **Unsuccessful treatment:** a treatment that end up in treatment default or loss to follow up, treatment failure or death.

The extracted data were checked for consistency and completeness and entered in to Epinfo version 7 that later exported to STATA 12 [23] for analysis. Data were described separately for the two groups (6EH and 4RH) using frequencies, mean, standard deviations and tables. Besides, crude comparisons of the baseline and follow up measures among the 6EH and 4RH groups were made using chi square (*x* ^*2*^) or t-tests as appropriate. Subsequently, bivariate and multiple binary logistic regression analysis were made to compute crude and adjusted odds ratios respectively between the explanatory and outcome variables. Multiple logistic regression model was fitted with those variables having *p* ≤ 0.2 on bivariate analysis. Finally, statistical significance was judged at *p* < 0.05 and/or 95 % confidence interval (CI) of odds ratio (OR) excluding one.

#### Ethical considerations

We received ethical approval from the institutional review board of the College of Health Sciences at Addis Ababa University. Accordingly, anonymous patient data were extracted from routine service registry upon permission from the respective institutions.

## Results

### Demographic and baseline clinical characteristics

We retrieved 846 patient records of which 790 (93.4 %) with complete outcome records [395 each treated with 2RHZE/6HE and 2RHZE/4RHregimens respectively] were described and analyzed. The mean age of the patients was 30.8 (31.5 vs 30.9 years, *p* = 0. 5) respectively, for those treated with 6HE and 4HR (Table 1). More than half, 56.6 % and 55.7 % of the patients were male and reside in rural areas, respectively, with no statistically significant difference among the two groups. With regard to the patient profile, 86.8 % and 77%were registered at health center and had pulmonary TB, respectively. Of the patients, 765 (96.8 %) were new cases (378 treated with 6EH vs387 with 4RH regimen) and the rest ((25 (3.2 %) (17 from 6EH vs 8 from 4RH) were transferred in and other cases treated with new case regimen. Human Immunodeficiency virus (HIV) test result was available for 612 (77.5 %) with statistically significant difference among the two groups [283 (71.6 %) from 6EH and 329 (83.3 %) from 4RH, *p* < 0.001]. Among those tested HIV positives, 44 (57.1 %) received either Cotrimoxazole Prophylactic Therapy (CPT) or Antiretroviral Therapy (ART) with no statistically significant difference among the regimens 24 (64.9 %) from 6EH and 20 (50 %), *p* = 0.2.

**Table 1:** Demographic and clinical characteristics of the tuberculosis patients registered between, 2008–2014, Southwest Ethiopia

Variable		Continuation phase treatment regimen		Total	*P* value
		6EH (*n* = 395) *n* (%)	4RH (*n* = 395) *n* (%)		
Age (years)	Mean ± SD^a^	31.1 ± 12.9	30.5 ± 11.9	30.8 ± 12.4	0.5
Gender	Male	221 (55.9)	226 (57.2)	447 (56.6)	0.7
	Female	174 (44.1)	169 (42.8)	343 (43.4)	
Residence	Urban	175 (44.3)	175 (44.3)	350 (44.3)	1
	Rural	220 (55.7)	220 (55.7)	440 (55.7)	
Zone	Kaffa	106 (26.8)	104 (26.3)	210 (26.6)	0.9
	Bench Maji	206 (52.2)	203 (26.3)	409 (51.8)	
	Sheka	83 (21.0)	88 (22.3)	171 (21.6)	
Treatment center	Hospital	55 (13.9)	49 (12.4)	104 (13.2)	0.5
	Health center	340 (86.1)	346 (87.6)	686 (86.8)	
Baseline weight	Mean ± SD^a^	47.6 ± 8.6	48.4 ± 8.5	48 ± 8.5	0.2
Baseline sputum smear	Positive	186 (47.1)	173 (43.8)	359 (45.4)	0.6
	Negative	151 (38.2)	165 (41.8)	316 (40)	
	Unknown	58 (14.7)	57 (14.4)	115 (14.6)	
Type of TB	Pulmonary	303 (76.7	305 (77.2)	608 (77.0)	0.9
	Positive	186 (47.1)	173 (43.8)	359 (45.4)	
	Negative	117 (30.4)	132 (33.4)	252 (31.9)	
	Extra pulmonary	92 (23.3)	90 (22.8)	179 (23)	
HIV status	Positive	37 (9.4)	40 (10.1)	77 (9.7)	<0.001
	Negative	246 (62.3)	289 (73.2)	535 (67.7)	
	Unknown	112 (28.4)	66 (16.7)	178 (22.5)	
Received CPT^b^ (*n* = 77)	Yes	18 (48.6)	17 (42.5)	35 (45.5)	0.6
Received CPT or ART^c^ (*n* = 77)	Yes	6 (16.2)	6 (15.0)	12 (15.6)	0.8
Received ART (*n* = 77)	Yes	12 (32.4)	9 (22.5)	21 (27.3)	0.3
Baseline sputum smear	Positive	186 (47.1)	173 (43.8)	359 (45.4)	0.6
	Negative	151 (38.2)	165 (41.8)	316 (40)	
	Unknown	58 (14.7)	57 (14.4)	115 (14.6)	

^a^Standard deviation,^b^ *CPT* Cotrimoxazole prophylactic therapy, ^c^ *ART* antiretroviral therapy

### Patient follow-ups and treatment outcomes

A total of 695 (88 %) of the patients had successful treatment outcomes with statistically significant difference (85.3 % vs 90.6 %, *p* = 0.02) among the 6HE and 4RH groups, respectively (Table 2). A total of, 324 (90.3 %), 208 (85.4 %) and 163 (91.1 %) of pulmonary positive, pulmonary negative and extra pulmonary TB cases respectively had successful outcomes with statistically significant difference, *P* = 0.03. Besides, statistically significant differences in successful outcomes, 64 (83.1 %), 482 (90.1 %) and 149 (83.7 %, *p* = 0.005 were also found among HIV positive, HIV negatives and unknown HIV status TB cases, respectively.

**Table 2:** Patient follow-up measures and treatment outcomes of TB patients registered during 2008–2014, Southwest Ethiopia

Variables		Continuation phase treatment regimen			
		6EH (*n* = 395) *n* (%)	4RH (*n* = 395) *n* (%)	Total *N* (%)	*P* value
Weight at 2^nd^ month	Mean ± SD	49.9 ± 8.9	50.8 ± 8.2	50.4 ± 8.6	0.2
Weight at 5^th^ month	Mean ± SD	51.4 ± 8.6	51.3 ± 7.3	51.3 ± 7.9	0.9
Weight at 6/7^th^ month	Mean ± SD	51.1 ± 8.9	52.5 ± 6.5	51.7 ± 7.9	0.3
Change in weight at 2^nd^ month	Not increased	49 (12.4)	61 (15.4)	110 (13.9)	0.4
	Increased	191 (48.4)	177 (44.8)	368 (46.6)	
	Unknown	155 (39.2)	157 (39.7)	312 (39.5)	
Sputum smear end of 2^nd^ month (*n* = 359)	Positive	4 (2.2)	2 (1.2)	6 (1.7)	0.09
	Negative	130 (69.9)	138 (79.8)	268 (74.7)	
	Unknown	52 (28)	33 (19.1)	85 (23.7)	
Sputum smear end of 5^th^ month (*n* = 359)	Positive	1 (0.5)	0 (0)	1 (0.3)	0.03
	Negative	83 (44.6)	100 (57.8)	183 (51)	
	Unknown	102 (54.8)	73 (42.2)	175 (48.7)	
Sputum smear end of 6/7^th^ month (*n* = 359)	Positive	0	0	0	0.8
	Negative	94 (50.5)	85 (49.1)	179 (49.9)	
	Unknown	92 (49.5)	88 (50.9)	180 (50.1)	
Sputum smear done during treatment (*n* = 359)	No	44 (23.7)	33 (19.1)	77 (21.4)	0.3
	At least once	142 (76.3)	140 (80.9)	282 (78.6)	
Continuation phase visit	Mean ± SD	5.8 ± 1.1	5.4 ± 3.1		0.09
Treatment outcome	Successful	337 (85.3)	358 (90.6)	695 (88)	0.02
	Cured	77 (19.5)	85 (21.5)	162 (20.5)	
	Completed	260 (65.8)	273 (69.1)	533 (67)	
	Unsuccessful	58 (14.7)	37 (9.4)	95 (12)	
	Died	28 (7.1)	18 (4.6)	46 (5.8)	
	Defaulted	29 (7.3)	19 (4.8)	48 (6.1)	
	Failure	1 (0.3)	0	1 (0.1)	

Measurements of patient weight at the end of second, fifth and sixth/seventh months of treatment were available for 504 (63.8 %), 145 (18.4 %) and 141 (17.8 %) respectively. Thus, 368 (46.6 %) or (48.4 % from 6EH and 44.8 % from 4RH, *p* = 0.4) have gained some amount of weight at the end of second month of treatment. On the other hand, of those initially smear positive pulmonary TB cases, 78.6 % had undergone sputum follow up examination at least once after the diagnosis (76.3 % among 6HE and 80.9 % among 4HR, *p* = 0.3). Thus sputum smear results at the end of second, fifth and sixth/seventh months of treatment were available for274 (76.3 %), 184 (51.3 %) and 179 (49.9 %) cases respectively with no statistically significant differences among the 6EH and 4RH regimens. The majority of the smear positives (69.9 % vs 79.8 % respectively from the 6EH and 4RH regimens, *p* = 0.4) converted to negative at the end of second month treatment.

### Factors associated with unsuccessful treatment outcomes

In bivariate analysis patient age, residence, zone, weight change at the end of the second month of treatment, sputum smear follow-up and continuation phase regimen are associated with treatment success at *p* < 0.05. But, in multivariate analysis 4RH continuation phase treatment regimen [AOR (95 % CI) 0.55 (0.34,0.89)], patient age [AOR (95 % CI) 1.02 (1.001,1.022)], rural residence [AOR (95 % CI) 2.1 (1.18,3.75)], treated at health center [AOR (95 % CI) 0.37 (0.14,0.97)], HIV positives [AOR (95 % CI 2.38 (1.12,5.07)], gained weight at the end of the second month [AOR (95 % CI) 0.28 (0.11,0.72)] independently predicted unsuccessful treatment outcome (Table 3). The odds of unsuccessful outcome was higher among the older, rural residents, HIV positives and unknown weight change at the end of second month treatment. The odds of having unsuccessful outcome increase by 2 % for every one year increase in age (AOR = 1.02).On the other hand, treated with 4RH continuation phase regimen, being treated at health center and weight gain at the end of second month have lower likelihood of unsuccessful outcome. Patients put on 4RH continuation phase of treatment regimen are 45 % less likely to have unsuccessful outcome compared to those put on 6EH regimen. Patients treated at health center have about 63 % lower odds of unsuccessful outcome as compared to those treated at hospitals. HIV co infected TB patients have more than two fold higher risk of unsuccessful outcomes compared to HIV negatives (AOR = 2.38). Those patients gained weight at the end of the second month of treatment have 72 % lower odds of unsuccessful outcomes compared to those with reduced or unchanged weight (AOR = 0.28). A subgroup analysis among smear positive pulmonary cases showed having a sputum checkup at least once during treatment independently predicted 96 % lower odds of unsuccessful outcomes compared to those unchecked (AOR 0.04 (95 % CI, 0.01–0.12), *P* < 0.001) (Additional file 1).

**Table 3:** Factors associated with unsuccessful treatment outcomes among TB patients registered during 2008–2014, Southwestern Ethiopia

Variables		Treatment outcomes		Odds ratio (OR)	
		Unsuccessful *n* (%)	Successful *n* (%)	Crude OR 95 % CI^a^	Adjusted OR 95 % CI
Age (years)	Mean(SD)	33.5 (14)	30.4 (12.0)	1.02 (1.002,1.03)	**1.02 (1.001,1.022)**
Gender	Male	60 (13.4)	387 (86.6)	1	1
	Female	35 (10.2)	308 (89.8)	0.73 (0.47.1.14)	0.63 (0.38,1.03)
Residence	Urban	32 (9.1)	318 (90.9)	1	1
	Rural	63 (14.3)	377 (85.7)	1.66 (1.06,2.61)	**2.1 (1.18,3.75)**
Zone	Kaffa	34 (16.2)	176 (83.8)	1	1
	Bench Maji	50 (12.2)	359 (87.8)	0.72 (0.45,1.14)	1.41 (0.73,2.75)
	Sheka	11 (6.4)	160 (93.6)	0.36 (0.17,0.73)	1.2 (0.44,3.32)
Treatment center	Hospital	17 (16.3)	87 (83.7)	1	1
	HC	78 (11.4)	608 (88.6)	0.66 (0.37,1.16)	**0.37 (0.14,0.97)**
Type of TB	Pulmonary	78 (12.8)	530 (87.2)	1	1
	EPTB^b^	17 (9.3)	165 (90.7)	0.70 (0.40,1.22)	0.57 (0.32,1.04)
HIV status	Negative	53 (9.9)	482 (90.1)	1	1
	Positive	13 (16.9)	64 (83.1)	1.85 (0.95,3.57)	**2.39 (1.12,5.07)**
	Unknown	29 (16.3)	178 (83.7)	1.77 (1.08,2.88)	**2.26 (1.23,4.11)**
Weight change end of 2^nd^ month	No increase	11 (8.2)	357 (91.8)	1	1
	Increased	9 (3)	101 (97)	0.35 (0.14,0.86)	**0.28 (0.11,0.72)**
	Unknown	75 (24)	237 (76)	3.55 (1.71,7.37)	**3.48 (1.60,7.54)**
Continuation phase regimen	6EH	58 (14.7)	337 (85.3)	1	1
	4RH	37 (9.4)	358 (90.6)	0.60 (0.39,0.93)	**0.55 (0.34,0.89)**

^a^Confidence interval, ^b^Extra pulmonary Tuberculosis, bold figures indicate statisticaly significant at *p*<0.05

## Discussion

Treatment outcomes among TB patients treated with RHZE for the first 2 months, followed by HE for 6 months (2RHZE/6EH) and RH for 4 months (2RHZE/4RH) was compared. Both groups of the cases had no statistically significant difference with respect to socio-demographic, baseline clinical, bacteriologic and follow up measures that depict comparability of the groups. The comparison was made between regimens used during the continuation phase treatment (4RH vs 6EH). Thus a lower rate of unsuccessful outcomes was reported among those treated with 4RH continuation phase regimen.

A statistically significant difference in treatment outcomes where lower unsuccessful treatment outcome (9.4 % vs 14.7 %) was observed among patients treated with 4RH and 6EH regimen respectively. Similarly, a study conducted in Nigeria [14] reported higher odds of unsuccessful outcome among those treated with 6EH. This could be due to the differences in length and type of drugs used during the continuation phase treatment those influence adherence and ultimate outcome. Studies reported that reduced continuation phase (from 6EH to 4HR) treatment is associated with lower cost and expected mortality [12] that enhance successful treatment outcome. On the other hand, use of rifampcin for longer period of time during the treatment of TB is associated with better outcomes [13] that might be related with efficacy of the drug. The finding implies the adoption [11] of the latest WHO recommendation [7] in high prevalent and resource constrained settings is working well.

Apart from the treatment regimens, patient attribute like age and residence independently predicted treatment outcomes. We found that age had an inverse relation with unsuccessful outcome where the odds of unsuccessful outcome increase with age. Several studies also reported that older patients were more likely to have unsuccessful outcomes than younger [24–27]. This could be due to higher risk of age related co morbid situations those lead to poor adherence and outcomes [28]. Patients residing in rural areas had higher risk of unsuccessful outcomes which could be attributed to the low access to TB care and unfavorable living conditions. The findings imply need for focused intervention targeted to those older age groups and rural dwellers besides the treatment regimen.

Monitoring of patient weight and sputum are among the recommended follow-up measures required for TB patients on treatment [7]. The results of both weight and sputum monitoring are used to adjust for drug dose and predict outcomes of the treatment. The proportion of smear positive patients converted to negative at the end of the intensive phase has been taken among indicators of TB programme performance [7]. However, only small proportion of patients had documented results of the weight and sputum follow-ups particularly at the later periods of treatment. Consistent to findings from African settings [29] majority of those patients undergone sputum checkup during treatment converted to negative.

Patients gained some amount of weight at the end of second month treatment had lower risk of unsuccessful outcomes which is consistent with other studies [30, 31]. This could be explained by the fact that weight gain marks some level of improvement from the TB illness including reduced appetite. In addition, changes in weight while on treatment might be an indication of appropriateness of the drug dose to treat the illness. On the other hand those with unknown weight change at the end of first two months treatment had higher odds of unsuccessful outcomes. This might have occurred due to possible misclassification of cases with reduced or remains unchanged to unknown. The weight change might be unknown due to patients’ treatment interruption subsequent to treatment default or death those constitute unsuccessful outcome. So that patient’s status of weight during treatment might be left undetermined.

Having sputum checkup at least once during treatment among initially smear positives predicted lower risk of unsuccessful outcome. However, reviews showed low sensitivity and modest specificity of sputum results at the end of intensive phase to predict failure and relapse [32]. On the other hand, detection of sputum positive during treatment trigger further patient assessment that influence treatment regimens and ultimate outcome. Hence, the routine sputum monitoring adopted by the country [11] during treatment should be improved as it is an indicator of program performance and trigger for patient assessment.

Consistent with other studies [33, 34], HIV positive TB patients are more likely to have unsuccessful outcomes compared to those negatives. This could be due to multifaceted influences of HIV on TB diagnosis and response to TB treatment those negatively affect the outcomes of TB treatment [35]. Consequently, collaborative services have been recommended in order to curb the influence of HIV on TB and vice versa [36]. Evidences from systematic review in African context supported the recommendation and reported better outcomes among concurrently screened and managed TB and HIV infected patients [37]. Nonetheless, we found no statistically significant difference in treatment success among those infected TB patients provided with CPT and/or ART. The indifference could be explained by the low uptake of integrated TB/HIV collaborative services among the studied patients. In this study, more than three quarter of the patients were offered HIV test which is little higher than the national average of 65 % [18] and 9.7 % TB/HIV co infected patients which is almost similar to the national average of 10 %. However, only few HIV co infected patients (45 % on CPT and 27.3 % on ART) were found to have documented service provision. [18]. The discrepancies could be due to differences in reporting periods where the national average is a single year attainment but that of this study is over a period of six years including the nationally reported year. Over all, the targets set for the TB/HIV service collaboration has not yet met which calls for in depth understanding and focused intervention that suit local settings. On the other hand, unknown HIV status predicted higher odds of unsuccessful outcomes. This could be due to possible misclassification of HIV positive cases those predict worse outcome in to unknown. The study is limited to control for changes in medical resources, polices and quality of care across the study periods. Since only few variables were captured on the register, we could not control for possible confounding effect of socioeconomic, lifestyle and co morbid illness. Furthermore resistance pattern of the treatment regimens could not be evaluated which is recommended for the assessment of impact of treatment regimens. On the other hand the random selection of relatively large sample from both groups minimized risk of selection bias. The groups treated with 4RH and 6EH had insignificant differences with regard to baseline and follow-up clinical and bacteriologic attributes that enhanced comparability of the groups. Besides, we extracted data from a standardized routine programme register that reflect operational reality. In general, our study is valid and can apply in similar settings given the limitations.

## Conclusion

In conclusion, the switch of continuation phase TB treatment regimen for new cases from 6EH to 4RH has brought better treatment outcomes. The findings verified the applicability of latest WHO recommendation and national adoption to the high prevalent and resource constrained settings. However, the unsuccessful outcome among the older, rural dwellers and HIV positives is higher independent of the treatment regimen that need further investigation and focused intervention. Therefore, the recommended switch of treatment regimen should be maintained and progressively assessed for outcomes, including drug resistance survey or surveillance. Moreover, further studies should be carried out on the impact of treatment regimens among older, rural residents and HIV positives.

## Additional file

Additional file 1: Table 4: Factors associated with unsuccessful treatment outcome among smear positive pulmonary TB cases registered during 2008-2014, southwestern Ethiopia (n=359). Table 5: Factors associated with unsuccessful outcome among clinically diagnosed (smear negative 10.1186/s12879-016-1917-0 pulmonary and extra pulmonary) TB cases registered during 2008-2014, Southwestern Ethiopia (n=431). (DOCX 17 kb)

## Abbreviations

ART: Antiretroviral therapy
CI: Confidence interval
CPT: Cotrimoxazole prophylactic therapy
DOTS: Directly Observed Treatment Short course
EH: Ethambutol and Isoniazid combination
HIV: Human Immunodeficiency Virus
OR: Odds ratio
RH: Rifampicin and Isoniazid combination
RHZE: Rifampicin, Isoniazid, Pyrazinamide and Ethambutol combination
TB: Tuberculosis
WHO: World Health Organization
