# Supplementary material for: Tuberculosis treatment outcomes of six and eight month treatment regimens in districts of Southwestern Ethiopia: a comparative cross-sectional study
Source: BMC Infect Dis. 2016 Nov 8;16:653. doi: 10.1186/s12879-016-1917-0 (PMC5100220; doi:10.1186/s12879-016-1917-0)
Supplement: Additional file 1: — Table 4: Factors associated with unsuccessful treatment outcome among smear positive pulmonary TB cases registered during 2008-2014, southwestern Ethiopia (n=359). Table 5: Factors associated with unsuccessful outcome among clinically diagnosed (smear negative 10.1186/s12879-016-1917-0 pulmonary and extra pulmonary) TB cases registered during 2008-2014, Southwestern Ethiopia (n=431). (DOCX 17 kb) [file 12879_2016_1917_MOESM1_ESM.docx]

|  | | Treatment outcome | | Odds Ratio (OR) | |
| --- | --- | --- | --- | --- | --- |
| Variable | | Unsuccessful | Successful |  |  |
|  | | N(%) | N(%) | Crude OR(95% CI) | Adjusted OR(95% CI) |
| Age (mean/SD) years | | 30.7(12.5) | 30.3(11.7) | 1.002(0.97,1.03) | 0.99(0.95,1.05) |
| Gender | Male | 20(10.5) | 171(89.5) | 1.00 | 1.00 |
|  | Female | 15(8.9) | 153(91.1) | 0.84(0.41,1.70) | 0.52(0.21,1.30) |
| Residence | Urban | 9(7.0) | 120(93.0) | 1.00 | 1.00 |
|  | Rural | 26(11.3) | 204(88.7) | 1.70(0.77,3.75) | 2.9(0.92,9.19) |
| Treatment center | Hospital | 6(23.1) | 20(76.9) | 1.00 | 1.00 |
|  | Health center | 29(8.7) | 304(91.3) | 0.32(0.12,0.85) | **0.14(0.03,0.70)** |
| Continuation phase regimen | 6EH | 23(12.4) | 163(87.6) | 1.00 | 1.00 |
|  | 4RH | 12(6.9) | 161(93.1) | 0.53(0.25,1.10) | 0.58(0.23,1.42) |
| HIV result | Positive | 4(16.0) | 21(84.0) | 1.00 | 1.00 |
|  | Negative | 20(7.7) | 240(92.3) | 0.44(0.14,1.4) | 0.23(0.04,1.24) |
|  | Unknown | 11(14.9) | 63(85.1) | 0.92(0.26,3.19) | 0.77(0.14,4.2) |
| Sputum follow up after diagnosis | None | 28(36.4) | 49(63.6) | 1.00 | 1.00 |
|  | At least once | 7(2.5) | 275(97.5) | 0.05(0.02,0.11) | **0.04(0.02,0.12)** |
| Weight change end of 2^nd^ month | No increase | 4(8.2) | 45(91.8) | 1.00 | 1.00 |
|  | Increased | 7(3.5) | 191(96.5) | 0.41(0.12,1.50) | 0.23(0.05,1.03) |
|  | Unknown | 24(21.4) | 88(78.6) | 3.1(1.003,9.38) | 0.52(0.12,2.3) |

Table 4: Factors associated with unsuccessful treatment outcome among smear positive pulmonary TB cases registered during 2008-2014, southwestern Ethiopia (n=359)

Table 5: Factors associated with unsuccessful outcome among clinically diagnosed (smear negative pulmonary and extra pulmonary) TB cases registered during 2008-2014, Southwestern Ethiopia (n=431)

|  | | Treatment outcome | | Odds Ratio (OR) | |
| --- | --- | --- | --- | --- | --- |
| Variable | | Unsuccessful | Successful |  |  |
|  | | N(%) | N(%) | Crude OR(95% CI) | Adjusted OR(95% CI) |
| Age (mean/SD) years | | 35.2(15.8) | 30.5(12.3) | 1.03(1.01,1.05) | **1.03(1.01,1.05)** |
| Gender | Male | 40(15.6) | 216(84.4) | 1.00 | 1.00 |
|  | Female | 20(11.4) | 155(88.6) | 0.70(0.39,1.24) | 0.64(0.34,1.21) |
| Residence | Urban | 23(10.4) | 198(89.6) | 1.00 | 1.00 |
|  | Rural | 37(17.6) | 173(82.4) | 1.84(1.05,3.21) | 1.74(0.88,3.42) |
| Treatment center | Hospital | 11(14.1) | 67(85.9) | 1.00 | 1.00 |
|  | Health center | 49(13.9) | 304(86.1) | 0.98(0.48,1.99) | 0.67(0.28,1.63) |
| Continuation phase regimen | 6EH | 35(16.7) | 174(83.3) | 1.00 | 1.00 |
|  | 4RH | 25(11.3) | 197(88.7) | 0.48(0.25,0.92) | 0.57(0.31,1.05) |
| HIV result | Positive | 9(17.3) | 43(82.7) | 1.00 | 1.00 |
|  | Negative | 33(12.0) | 242(88.0) | 0.65(0.29,1.46) | 0.47(0.19,1.16) |
|  | Unknown | 18(173) | 86(82.7) | 1.00(0.41,2.4) | 0.87(0.32,2.32) |
| Weight change end of 2^nd^ month | No increase | 5(8.3) | 55(91.7) | 1.00 | 1.00 |
|  | Increased | 4(2.4) | 166(97.6) | 0.27(0.07,1.04) | **0.22(0.06,0.86)** |
|  | Unknown | 51(25.4) | 150(74.6) | 3.83(1.46,10.10) | **3.42(1.27,9.21)** |
